# The Antiapoptotic Effect of Curcumin in the Fibroblast of the Cochlea in an Ototoxic Rat Model

**Published:** 2018-09

**Authors:** Tengku-Siti-Hajar Haryuna, Agustinus-Hamonangan-Winston Purba, Farhat Farhat, Widayat Alviandi

**Affiliations:** 1 *Department of Otorhinolaryngology-Head and Neck Surgery, Faculty of Medicine, Universitas Sumatera Utara, Medan 20155, Indonesia.*; 2 *Department of Otorhinolaryngology-Head and Neck Surgery, Faculty of Medicine, Universitas Indonesia, Jakarta 10430, Indonesia.*

**Keywords:** Apoptosis, Curcumin, Cochlea, Gentamicin, Prevention, Rats.

## Abstract

**Introduction:**

This study aimed to show the potency of curcumin as an antiapoptotic agent that decreases the apoptotic index in the cochlea lateral wall in ototoxic rat models.

**Materials and Methods:**

A total of 24 *Rattus norvegicus* were divided into eight groups: Group 1 (control group), Group 2 (gentamicin (+)), Group 3 (gentamicin + curcumin 20 mg/day), Group 4 (gentamicin + curcumin 40 mg/day), Group 5 (gentamicin + curcumin 20 mg/day for 7 days), Group 6 (gentamicin + curcumin 40 mg/day for 7 days), Group 7 (curcumin 20 mg/day for 3 days + gentamicin), and Group 8 (curcumin 40 mg/day for 3 days + gentamicin). After the division, the rats were terminated in order to measure the apoptotic index using a terminal deoxynucleotidyl transferase (TdT) dUTP nick-end labeling (TUNEL) assay in the fibroblasts of the cochlea lateral walls. The data were analyzed using analysis of variance (ANOVA), and P<0.05 was used as the cut-off for statistical significance.

**Results:**

Administration of gentamicin showed significant differences (P<0.05) in the apoptotic index. Groups undergoing curcumin treatment at a higher dose (200 mg/kg bw) and the prevention groups showed significant differences compared with groups not treated with curcumin

**Conclusion:**

This study concluded that the apoptotic index can be decreased by curcumin and has a preventive benefit toward ototoxic rat models. The administration of curcumin depended on the dose and duration.

## Introduction

Aminoglycosides are a popular group of antibiotics with considerable antimicrobial efficacy and toxic side effects on the kidneys and inner ears. Gentamicin is one example of an aminoglycoside antibiotic. The damage inflicted by gentamicin on the kidneys is reversible, but damage to the inner ears is irreversible. The ototoxic side effects may become evident days or weeks after systemic application, and the damage is bilateral (1). Ototoxic effects caused by aminoglycoside are cochleotoxic and vestibulotoxic (1,2).

The incidence ototoxicity induced by aminoglycosides is increasing in developing countries compared with industrialized countries (1). Aminoglycoside exposure leads to oxidative stress and radical oxygen species (ROS) formation, which leads to an apoptotic signalling pathway (3). Gentamicin induces ROS formation in cellular system and leads to the opening of mitochondrial permeability, ROS, and cytokines that may cause cell death through apoptosis or necrosis in a dose-dependent manner (4). Hearing loss by aminoglycosides on the cellular level is caused through damage of the cochlea hair cells, but the biochemistry and molecular mechanism are still hard to understand (5).Curcumin is a popular herb in South East Asia, and is used as a natural food spice (6,7) because of its color stability and low toxicity (6). Curcumin is a potential natural compound that impacts on different pathways, intracellular components, and important enzymes (8). Research with rat models has shown that curcumin has antiapoptotic activity against indomethacin, marked by a decreased expression of caspase-3 (9). The aim of this research was to show the potential of curcumin as an antiapoptotic agent in obstructing the apoptotic index in the cochlea lateral wall in ototoxic rat models, which could be the basis of future clinical trials.

## Materials and Methods

*1. Experimental treatments in animal models*

This research used male rats, *Rattus norvegicus*, weighing 150–250 g. Thirty-two rats were used in this study, divided into eight groups of four rats. The curcumin given to the rats was extracted from Curcuma longa L. (turmeric). The level of the curcumin was (16.62 ± 0.14)% b/b, counted using a thin-layer chromatographic (TLC)-densitometric method. The curcumin doses varied from 100 mg/kg to 200 mg/kg, and all doses were suspended in carboxy methyl cellulose (CMC) 0.5% and were administered through a nasogastric tube. The rats were first anesthetized with 10 mg/kg of xylazine and 90 mg/kg of ketamine, which were administered via an intraperitoneal injection (10). 

After the rats were calmed following the effect of the prior injection, they were injected with 0.03–0.05 ml of 40 mg/ml gentamicin (11). The dose used was gentamicin 40 mg/ml 0.1 ml, injected into the anterosuperior tympanic membrane of the rats with the help of a microscope. The rats were terminated 18 hours after being given the gentamicin injection (12).

The samples drawn in this study were from rats of the same strain that were homogeneous with respect to gender and age, and were bred in the Biochemistry Laboratory, Faculty of Medicine, Airlangga University. Food for rats was provided *ad *libitum, and the room temperature in the laboratory was maintained between 20 and 26 °C. Lighting inside the cages during the light phase was maintained at an exposure below the reluctance threshold for rats, and the relative ambient humidity was 55±15%. Ethical permission to conduct this research was obtained from the Health Ethics Committee, Faculty of Medicine, University of North Sumatra/H. Adam Malik General Hospital, Indonesia. The ethics code was 433/KOMET/FK USU/2015.

The rats studied in this research were divided into eight groups. Group 1 was the control group which was not treated and was only given 5 ml of CMC; Group 2 was injected with gentamicin and was terminated 18 hours afterwards; Group 3 was injected with gentamicin, then after 18 hours, the rats were given 20 mg of curcumin as a single dose and were terminated 18 hours later; Group 4 was injected with gentamicin, then after 18 hours the rats were given 40 mg of curcumin as a single dose and were terminated 18 hours later; Group 5 was injected with gentamicin, then after 18 hours, the rats were given curcumin for 7 days at a dose of 20 mg per day and were terminated 18 hours after the final curcumin dose; Group 6 was injected with gentamicin, then after 18 hours they were given curcumin for 7 days at a dose of 40 mg per day, and were terminated 18 hours after the final curcumin dose; Group 7 was given 20 mg of curcumin per day for 3 days, then were injected with gentamicin 18 hours after the final curcumin dose, and were terminated 18 hours later; Group 8 was given 40 mg of curcumin per day for 3 days, then gentamicin injection 18 hours after they were given the last dose of curcumin, and were terminated 18 hours afterwards. Groups 3, 4, 5, and 6 were curative groups, in which curcumin was given after gentamicin. Groups 7 and 8 were preventive groups, in which curcumin was given before gentamicin. The rats were terminated and examined through necropsies. Temporal bone tissues from the head were taken and fixated with 10% formalin buffer solution and decalcified with ethylenediaminetetraacetic acid (EDTA) within 4 weeks. They were then tested in the laboratory to assess the apoptotic index on the fibroblasts of the cochlea lateral wall.

*2. Apoptosis examination*

The apoptosis examination was performed using an TACS 2 Tdt-DAB *in situ* apoptosis detection kit (Trevigen, Helgermen Ct. Gaithersburg). The procedures were performed through various steps. Samples were immerse hydrated, fixed, and immobilized in 1× phosphate-buffered saline (PBS) for 10 min, then the samples were covered with 50 μl of proteinase K solution for 15–30 min, and washed twice in deionized water for 2 min each time. The samples were immersed in quenching solution for 5 min, and washed in 1× PBS for 1 min. The samples were immersed in 1x Tdt labeling buffer for 5 min and covered with 50 μl of labeling reaction mix and incubated for 60 min at 37 °C in a humidity chamber. The samples were immersed in 1× Tdt stop buffer for 5 min, washed twice in deionized H_2_O, for 5 min each time. The samples were covered with 50 μl of Strep-HRP solution and incubated for 10 min at 37 °C in a humidity chamber to avoid evaporation. The samples were washed twice in 1× PBS, for 2 min each time, then immersed in 1% methyl green for 30 s up to 5 min. Slides were dipped 10 times each in two changes of deionized H_2_O, 95% and 100% ethanol. Each of them was dipped 10 times in two changes of o-xylene, with mount glass coverslips using mounting medium.The apoptotic index was analyzed using an Olympus XC 10 microscope under 40× magnification, marked by brown-stained nuclear staining, and counted in a masked manner using terminal deoxynucleotidyl transferase (TdT) dUTP nick-end labeling (TUNEL) (+). The researchers counted two randomly selected fields (13).

*3. Statistical analysis*

Data were analyzed by one-way analysis of variance (ANOVA) using IBM SPSS statistical software, with a significance level of 0.05.

## Results

**Fig 1:**
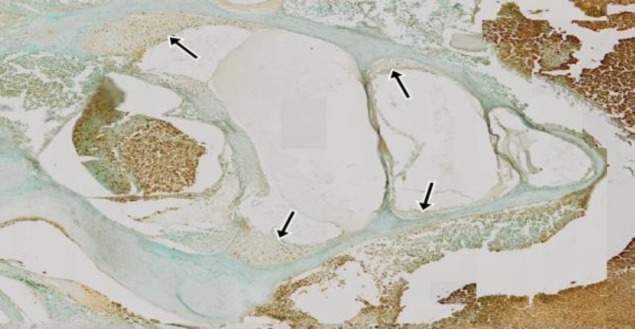
Supporting tissues and lateral cochlea wall with TUNEL assay (10× magnification). The arrows indicate the lateral wall

**Fig 2:**
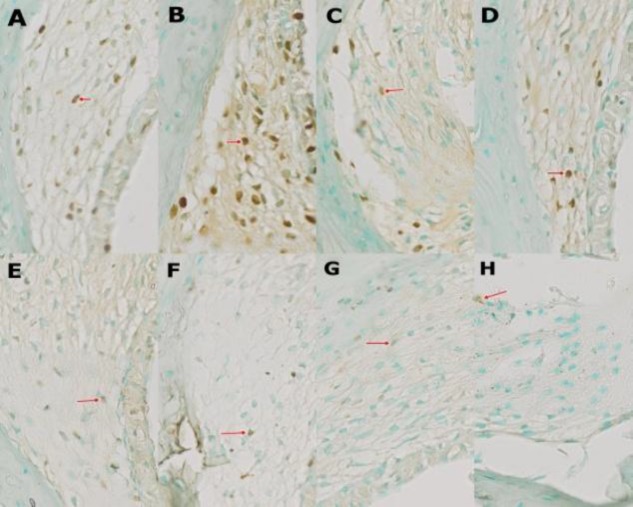
Apoptosis index in each group (40× magnification). (A) Group 1; (B) Group 2; (C) Group 3; (D) Group 4; (E) Group 5; (F) Group 6; (G) Group 7; (H) Group 8. Description: The arrows indicate TUNEL (+) (40× magnification)

  Figure 1 shows the lateral wall of the cochlea and Figure 2 shows the role of curcumin in the lateral wall of the cochlea of *Rattus norvegicus* gentamicin ototoxic models, observed using the TUNEL assay method, in which the researchers assessed the fragmentation of DNA in the cell nucleus that was brown.

**Fig 3:**
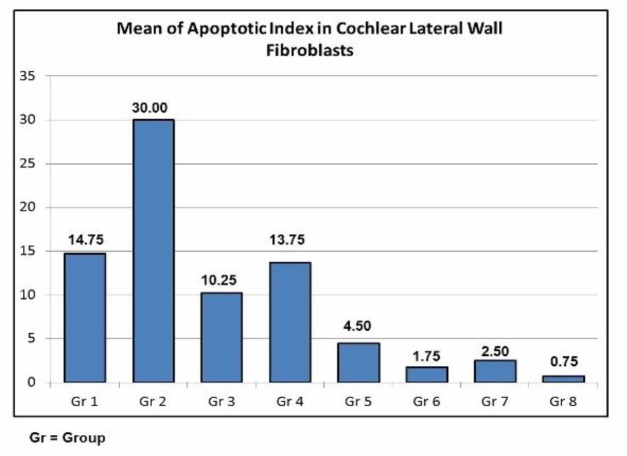
The Mean of Apoptotic Index in Cochlear Lateral Wall Fibroblasts

In Group 2, where the rats were only given gentamicin, their cell nuclei were mostly brown, compared with other groups (Groups 1,3-8). As shown in Figure 3, the highest average number of nuclei of cells undergoing apoptosis in the fibroblasts of the cochlea lateral walls was found in Group 2. The mean decrease in the number of core cells undergoing apoptosis in the fibroblasts of the cochlea lateral walls were found in all groups that received curcumin (Groups 3- 8). Table 1 shows significant differences (P<0.05) between the control group and the other treatment groups, except for Groups 3 and 4. The curcumin administration influenced the apoptotic index in all groups, compared with Group 2. Table 1 also shows that the dose and duration of the administration of curcumin affected the apoptotic index.

**Table 1:** ANOVA LSD test result in terms of apoptotic index

**Group**		**Mean difference ±Standard deviation**	**P-value**
1	2 3 4 5 6 7 8	−15.250 ± 3.433 4.500 ± 3.433 1.000 ± 3.433 10.250 ± 3.433 13.000 ± 3.433 12.250 ± 3.433 14.000 ± 3.433	0.000* 0.202 0.773 0.006* 0.001* 0.002* 0.000*
2	3 4 5 6 7 8	19.750 ± 3.433 16.250 ± 3.433 25.500 ± 3.433 28.250 ± 3.433 27.500 ± 3.433 29.250 ± 3.433	0.000* 0.000* 0.000* 0.000* 0.000* 0.000*
3	4 5 6 7 8	−3.500 ± 3.433 5.750 ± 3.433 8.500 ± 3.433 7.750 ± 3.433 9.500 ± 3.433	0.318 0.107 0.021* 0.033* 0.011*
4	5 6 7 8	9.250 ± 3.433 12.000 ± 3.433 11.250 ± 3.433 13.000 ± 3.433	0.013* 0.002* 0.003* 0.001*
5	6 7 8	2.750 ± 3.433 2.000 ± 3.433 3.750 ± 3.433	0.431 0.566 0.286
6	7 8	−0.750 ± 3.433 1.000 ± 3.433	0.829 0.773
7	8	1.750 ± 3.433	0.615

* Denotes statistical significance

## Discussion

The principal microscopic changes were found in the lateral wall spiral ligament, with degeneration of mitochondria, and location of endocochlear potential production. The research was performed by giving rats 3-nitropropionic acid directly into their round window membrane to evaluate the destruction in the cochlea lateral wall (14). This information encouraged the researchers to conduct research in the lateral wall of cochlea.

In this study, the researchers analyzed the antiapoptotic role of curcumin as seen from the index of apoptosis in the fibroblasts of ototoxic rat models. Giving the rats 40 mg/ml of gentamicin enhanced the apoptotic index in the lateral wall of the cochlea fibroblasts compared with the control group. This was also found in a study in Chinchillas that were given gentamicin and showed improvement in cochlea cell death markers, including cytochrome C, caspase-9 and caspase-3 (15). The same intratympanic gentamicin dose injection was once given to albino Wistar rats as controls to evaluate the effects of ototoxic intratympanic terbinafine in the middle ear (11). The rats were terminated 18 hours after the administration of gentamicin in order to follow the results of the round window membrane of Hartley strain guinea pigs by gelatin sponge soaked in gentamicin, where the representative photomicrograph showed TUNEL-positive nuclei, mostly in groups of 18 hours (12).

The results showed that the administration of curcumin decreased the apoptotic index in the lateral wall of the cochlea of ​the fibroblasts of rats that were seen in the comparison of Group 2 and Groups 3,4, 5, and 6. A similar finding was observed in the testicular tissues of the rats in which the administration of cadmium significantly increased testicular apoptosis. By administering curcumin, the reactivity and number of germinal cells of Leydig cells undergoing apoptosis was significantly reduced (16).

Administration of a dose of 100 mg/kg of curcumin once daily for 7 days has been proven to decrease the apoptotic index in rats (10). Therefore, the researchers in this study gave the rats curcumin for 7 days. In the groups in which rats were given a dose of 200 mg/kg of curcumin, the researchers investigated whether an increase in dose could give a better anti-apoptosis result. The same dose of curcumin was given to reduce the number of TUNEL-positive cells in rats that underwent partial hepatectomy (10).

The results show that the comparison of Groups 6 and 3 with other groups was statistically significant, which means that the provision of curcumin at larger doses and longer duration is better in lowering the apoptotic index. The comparison between Groups 5 and 6 and Group 4 was statistically significant, which means that curcumin administration with a longer duration is better in lowering the apoptotic index. A single dose of curcumin was sufficient for the preventive effect, as shown in the decreased the expression of caspase-3 that leads to the conclusion of antiapoptotic activity (9). In this research, curcumin was given for 3 days. The results showed that the ratio of the prevention groups (Groups 7 and 8) compared with Groups 1, 2, 3, and 4 showed statistically significant results, which means that curcumin has a preventive role in reducing the apoptosis index. This is consistent with a study in which rats were given acetaminophen, which reported 469% DNA fragmentation when compared with the control group (quantitative marker was considered the presumed predictor of apoptotic cell death), and decreased to 162% (almost 3×) in rats receiving curcumin as a preventive measure (7).

Apoptosis caused by aminoglycosides could occur through a number of mechanisms. First aminoglycosides may enter the cell by opening the cation channels directly in the cytosol. Aminoglycosides that bind to ferric iron (Fe^III^) (3,17) will then bond Fe^II^-aminoglycoside complexes in the cytosol and form ROS compounds, using arachidonic acid as a donor electron. ROS then activates Bax (protein pro-apoptotic), which then translocates to the membranes of the mitochondria, and apoptosis can be halted if the proteins and antiapoptotic agents (Bcl-2 and Bcl-XL) are successfully stopped. In the second possible mechanism, cytochrome C exits through the mitochondrial transitional pores made by a Bax-dependent mechanism, then activates caspase-9 and caspase-3 and leads to apoptosis. A third possible mechanism, a caspase-independent mechanism, involves the release of endonuclease G and AIF from the mitochondria to induce apoptosis. In the fourth mechanism, the aminoglycosides, which are in the ER lumen/endoplasmic reticulum, become attached to the climp-63, then induce oligomerization which enables the 14-3-3 protein, resulting in apoptosis signaling and/or lead to JNK being turned on and the translocation of the c-jun toward the nucleus. Finally, the fifth mechanism involves the transcription factor c-jun leading to the transcription of apoptotic genes followed by apoptosis (17). Caspase and Bcl-2 family proteins are important ingredients in apoptotic signaling. As a consequence, aminoglycoside ototoxicity causes activation of caspase-9-induced cytochrome C released from the mitochondria to the cytosol. Caspase-9 activates caspase-3 (caspase executioner), which divides antiapoptotic proteins or block deoxyribonucleases and leads to cell death. The Bcl-2 family of proteins is made up of antiapoptotic Bcl-2 and Bcl-XL, namely the pro-apoptotic Bax and Bak. When apoptotic signals beat the inhibition/protection by Bcl-2 and Bcl-XL, Bax translocates from the cytosol to the mitochondria, and the mitochondria is released from cytochrome C, which then activates caspase-9. It is not clear how Bax triggered the release of cytochrome C, but it seems to involve the compilation of the mitochondrial permeability transition pore (17). The role of curcumin in anti-apoptosis indicated by the decreased expression of Bax (12;18) in the ratio of Bax/Bcl-2 (18,19) decreased the activity of caspase-3 (18,19) as well as a decrease in the activity of caspase-9 (18), increased the concentration of the protein Bcl-2 (18) and the expression of Bcl-Xl (7).

In other research, curcumin has a protective effect against noise-induced hearing loss (NIHL), which is hypothesized to be through the inhibited the degradation of Ikβ in the cochlea and suppression formation of 4-hyroxynonenal (4-HNE) in the cochlea spiral ligament, which leads to suppression of the NFκβ signal (20). In vivo research demonstrated the protective effect of curcumin against cisplatin-induced ototoxicity. The mechanism is not yet clear, but curcumin acts as direct scavenging activity and indirect anti-oxidant action by induction of endogenous heme oxygenase-1 (HO-1) (21).

## Conclusion

Biomolecular curcumin has been proven to lower the index of apoptosis in a rat model of ototoxic due to gentamicin. This research showed that curcumin is capable of counteracting the ototoxic effects from the application of gentamicin in the lateral wall of the cochlea in​​ the rats studied.
